# Eradication of metastatic mouse cancers resistant to immune checkpoint blockade by suppression of myeloid-derived cells

**DOI:** 10.1186/2051-1426-2-S3-P267

**Published:** 2014-11-06

**Authors:** Kibem Kim, Andrew Skora, Zhaobo Li, Ada Tam, Luis Diaz, Nickolas Papadopolous, Lee Blosser, Kenneth Kinzler, Bert Vogelstein, Shibin Zhou

**Affiliations:** 1 Medical Scientist Training Program, Ludwig Center for Cancer Genetics and Therapeutics, Sidney Kimmel Comprehensive Cancer Center, The Johns Hopkins University School of Medicine, Baltimore, MD, USA; 2 Ludwig Center for Cancer Genetics and Therapeutics, Sidney Kimmel Comprehensive Cancer Center, The Johns Hopkins University School of Medicine, Baltimore, MD, USA; 3 Department of Oncology, Sidney Kimmel Comprehensive Cancer Center, The Johns Hopkins University School of Medicine, Baltimore, MD, USA

## 

Recent clinical trials have shown highly promising responses in a subset of patients treated with immune checkpoint inhibitory anti-programmed cell death-1, anti-programmed cell death ligand-1 (PD-1), and anti-cytotoxic T-lymphocyte-associated antigen-4 antibodies (CTLA-4) [1-4]. However, immunotherapy against poorly immunogenic cancers remains a challenge. Large, modestly immunogenic CT26 tumors or poorly immunogenic metastatic 4T1 tumors in mice were unresponsive to anti-PD-1 and anti-CTLA-4 treatments. Co-treatment with DNA methyltransferase and HDAC inhibitors, and checkpoint inhibitors markedly improved treatment outcomes, curing more than 80% of the tumor-bearing mice. Functional studies revealed that the primary targets of the epigenetic modulators were myeloid-derived suppressor cells (MDSCs). In addition, reduction of MDSCs using antibodies directed against them or a PI3K inhibitor that reduced circulating MDSCs had similar antitumor effects to those observed with the epigenetic modulators. Our results show that elevated myeloid-derived suppressor cells (MDSCs) are responsible for the resistance to checkpoint inhibitors and that elimination of MDSCs can lead to cures of experimental, metastatic tumors.
